# The Role of Targeted Nutrition Education of Preschoolers and Caregivers on Sustained Consumption of Biofortified Orange-Fleshed Sweetpotato in Kenya

**DOI:** 10.1093/cdn/nzab096

**Published:** 2021-07-12

**Authors:** Sylvester O Ojwang, David J Otieno, Julius J Okello, Rose A Nyikal, Penina Muoki

**Affiliations:** Department of Agricultural Economics, University of Nairobi, Nairobi, Kenya; Department of Agricultural Economics, University of Nairobi, Nairobi, Kenya; International Potato Center, Kampala, Uganda; Department of Agricultural Economics, University of Nairobi, Nairobi, Kenya; International Potato Center, Nairobi, Kenya

**Keywords:** nutrition education, OFSP consumption, vitamin A deficiency, preschoolers, caregivers, Kenya

## Abstract

**Background:**

Persistent prevalence of high malnutrition in poor households in developing countries calls for enhancement of cost-effective nutrition interventions among the vulnerable groups. One responsive way is to promote regular consumption of home-grown biofortified foods, particularly in the micronutrient-deficient groups. Previous nutrition interventions have targeted adults with behavior change education, but have rarely explored the potential of nutrition education of preschoolers as change agents.

**Objectives:**

This study sought to assess the effect of nutrition education targeting preschool children and their caregivers on their consumption of vitamin A–biofortified orange-fleshed sweetpotato (OFSP) in rural farm households in Homa Bay County, Kenya.

**Methods:**

A total of 431 preschooler-caregiver pairs from 15 village-level clusters were recruited into a randomized controlled trial. The sample was randomized into 1 control (3 villages) and 3 treatment groups (4 villages each). Treatments involved channeling nutrition education to preschoolers through their learning materials (preschooler treatment); the caregivers through their mobile phones (caregiver treatment); and to both preschoolers and their caregivers simultaneously (integrated treatment). Baseline and follow-up household-level surveys were conducted with the caregivers, and consumption data were collected from the preschoolers using a child dietary diversity register. Class teachers sought 24-h consumption recalls of the preschoolers for 19 consecutive schooldays.

**Results:**

The results of a zero-inflated Poisson regression showed that the phone-mediated and multichanneled nutrition education approaches significantly increased the number of days of OFSP consumption. The integrated nutrition education approach significantly increased the preschoolers’ likelihood to consume OFSP, number of OFSP consumption days, and likelihood to consume it more than once per week by 11%, 77%, and 20%, respectively.

**Conclusions:**

Nutrition education through OFSP-branded preschoolers' learning materials and phone-mediated messages provides effective nudges to the caregivers to feed their preschoolers regularly with OFSP. This could have implications for realizing sustainable nutrition programs in biofortified crop-growing areas.

## Teaser

A field trial in rural Kenya found that an integrated nutrition education intervention involving the preschoolers and caregivers’ phone-mediated text messages significantly increased biofortified orange-fleshed sweetpotato consumption days in preschoolers.

## Introduction

Diet-related health challenges are stubbornly rising with global population growth (1). Micronutrient deficiencies are reported to be more prevalent in the sub-Saharan Africa (SSA) countries, with anemia ranging from 46% to 71%, vitamin A deficiency (VAD) in under-5-y-olds at 48%, iodine deficiency at 36%, and zinc deficiency at 25% (2). These have devastating effects on the socioeconomic welfare of nations and societies. For instance, the cost of child undernutrition in Kenya was estimated to be 6.9% of the country's gross domestic product in 2014 (3). Underlying the deficiencies are unsustainable and insufficient nutrient intakes due to consumption of nutrient-poor foods and low dietary diversity. Conventional micronutrient malnutrition eradication approaches such as vitamin and mineral supplementation and fortification of processed food, although effective, have proved unable to provide a holistic solution to these malnutrition situations in all socioeconomic settings. This is particularly true in the case of rural poor and/or marginalized societies. However, biofortification of food staples has since been recognized as a significant food systems approach toward delivering sustainable, high-quality, and affordable diets to alleviate malnutrition problems (1, 4).

The vitamin A–biofortified orange-fleshed sweetpotato (OFSP) is now the leading success story, in terms of empirical evidence, of biofortification as an effort to combat micronutrient deficiencies (5). Studies have shown that daily consumption of 100–125 g boiled OFSP roots induced significant increments in the vitamin A content in the liver of children aged <5 y (6–9). Efficacy studies have also demonstrated that consumption of OFSP significantly reduced childhood diseases such as diarrhea in young children (10). Neela and Fanta (11) and another study in Kenya (12) ranked OFSP as the highest performing vegetable from a dietary and nutritional point of view, and specifically in terms of proximate, mineral, and β-carotene composition. Sweetpotato is a popular crop in western Kenya, where there is a rich tradition of its production for subsistence and income generation. However, the sustainable availability of OFSP on the menus of the households is considerably weak despite its short production period (12). This suggests the need to streamline OFSP campaigns to focus on ensuring its increased consumption per capita and not just replacing the white- and yellow-fleshed sweetpotato varieties with the OFSP on the menus.

Previous studies have repeatedly affirmed that the OFSP is popular among young children due to its attractive deep-orange color (11, 13), but consumption tends to be concentrated around harvest time due to limited supply (14). Sustained regular intake is desired to supply the vitamin A needs of the vulnerable groups. Although OFSP promotion campaigns have recommended its daily consumption (14), the rates are low in many countries. In most of the reviewed literature, a large proportion of the rural farming households consumed it on <4 d in the previous 7 d, for instance, 49.5% (*n* = 295) of rural households in Eastern Province in Zambia (15). Nurturing a dietary habit involving daily consumption of OFSP might also have a ripple effect on its supply due to the increased demand, thereby enhancing the food security status of the household through improved availability, access, and utilization of the nutritious food.

Nutrition education interventions have been applied in many behavior change communication projects in SSA with great promise of significant improvements in the nutritional status of the target households (16–18). However, these have mostly targeted adults. Recent projects have shown the potential of involving school children as change agents in the behavior change communication interventions to improve the production and intake of nutritious foods (19–22). Many of these projects have engaged children at primary school levels and involved school gardening and school feeding programs. To the best of our knowledge, the potential of nutrition education interventions involving preschoolers as change agents through the Early Childhood Development (ECD) and targeting increased home consumption of OFSP to reduce VAD is a significant gap in literature and policy. This study aimed to assess the effects of ECD-channeled nutrition education approaches involving preschoolers and caregivers on OFSP consumption. The specific objectives were to:

1. Assess the effects of targeted nutrition education approaches with preschoolers and caregivers on the number of OFSP consumption days in preschoolers.
2. Assess the effects of targeted nutrition education approaches with preschoolers and caregivers on the likelihood of consuming OFSP on >1 d/wk.

## Methods

### Study area and sampling

The experiment was done in one of the leading sweetpotato–producing counties in Kenya, Homa Bay County. The International Potato Center (CIP) implemented a large project that promoted production and use of OFSP along the value chain in the area under the Accelerated Value Chain Development program in the county. The county has a stunting prevalence of 26% in the under-5-y-olds (23). The study was implemented in 2 subcounties (Ndhiwa and Rangwe) where CIP promoted the OFSP project. It employed a multistage sampling procedure. In the first stage, the 2 subcounties were purposively selected. The second stage involved a purposive selection of villages that had not been reached with OFSP promotion campaigns to participate in the study. This resulted in the selection of 15 villages—7 in Ndhiwa subcounty and 8 in Rangwe. Third, routine OFSP promotion activities were implemented at 1 publicly managed ECD in each of the selected villages targeting caregivers of preschool children. These involved OFSP cooking demonstrations in February 2018 and the distribution of free vines (200 thirty-centimeter-long cuttings to each caregiver) in April 2018. A list of households that participated in the activities formed the sampling frame (*n* = 721). The study followed McConnell and Vera-Hernández (24) to calculate the sample size of the cluster-level randomized controlled study with an unequal number of clusters (15 clusters in 4 study groups). The formula suggested an average of 98 households in each of the 4 study groups, which yielded a total sample size of 431 after adjustment for a possible nonresponse rate of 10%. Consequently, a random sample of 431 preschooler-caregiver pairs was drawn using a probability proportionate to size sampling method from the 15 villages.

### Experimental design and interventions

**FIGURE 1:**
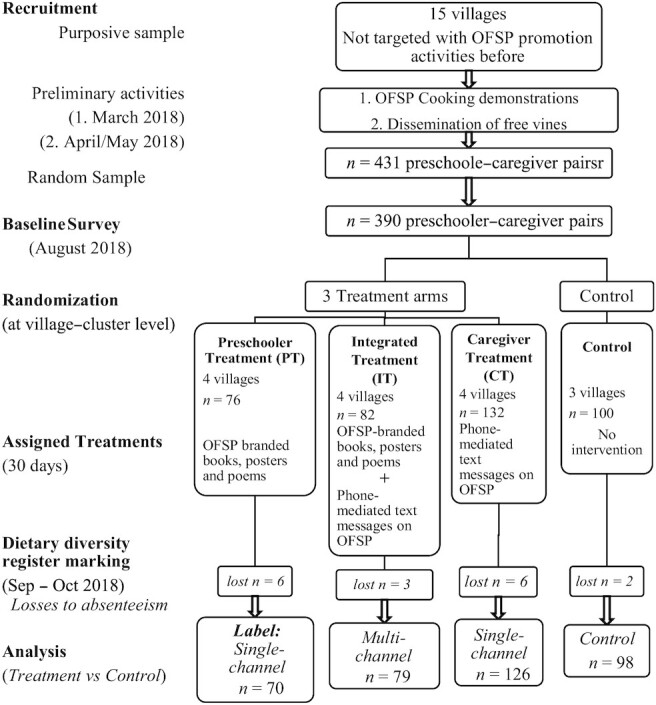
CONSORT diagram for the study: study design, activity protocol, and details. The left side of the chart shows the main activities in order of execution from top to the bottom, from the early stages involving recruitment of participants to the final stages describing the data presented for analysis. The details of the activities are presented in the corresponding boxes to the right. OFSP, orange-fleshed sweetpotato.

The study followed a cluster randomized controlled trial design. The randomization took place after the baseline survey with the caregivers in August 2018. The 15 villages, as clusters of the preschooler-caregiver pairs, were randomly assigned to 1 control arm (3 villages) and 3 treatment arms (4 villages in each) of the experiment. The treatment groups were subjected to nutrition education interventions for 30 d after the baseline survey. However, the control group was not assigned any intervention during this time. The CONSORT diagram in **Figure 1** summarizes the sampling procedure and the study design. The treatments were executed as follows.

#### Preschooler treatment

The preschooler treatment (PT) arm involved issuing OFSP-branded exercise books, class posters, and poems to preschool children alone. A total of 5 related messages were passed via the texts and pictorial illustrations on posters and book covers. The texts were in the local language (*Dholuo*), and the poem was in the English language (**Supplemental Figures 1** and **2**). The messages provided education on the identification and health benefits of OFSP to a preschooler and their family. Each preschooler received an exercise book, and the teachers displayed the 5 class posters and helped them to read the texts and recite the poems every school day. The goal was to see the preschoolers, as change agents, deliver the messages to their caregivers and thus nudge their decision to grow and consume OFSP.

#### Caregiver treatment

The caregiver treatment (CT) arm involved the issuance of OFSP-focused phone-mediated text messages to each caregiver while their preschoolers got no treatment. Again, the messages informed about identification, production, and health benefits of OFSP to the preschoolers and the caregivers. One of the 7 messages was sent uniformly in a day to all the targeted caregivers. Seven different messages were sent in 7 d and repeated over the 30 d of intervention. The messages were sent to the Communication Authority of Kenya for verification and approval before they were deployed to the caregivers as per the laws of Kenya (25).

#### Integrated treatment

The integrated treatment (IT) involved issuing the activities in the PT and CT approaches to an individual household simultaneously. That is, the caregiver got the phone-mediated text messages, while the preschooler of the same household got the OFSP-branded books, posters, and poems.

The interventions were issued for 30 d from the second week of September 2018. It was expected that during this time all the households were harvesting the OFSP, planted in April the same year. Consumption of OFSP was expected during this period, and the interventions were complementary to the routine nutrition education issued during vines dissemination and cooking demonstration sessions.

### Instruments and data collection

Data were collected using 2 tools; pretested questionnaires administered by trained enumerators to the caregivers, and dietary diversity registers marked by class teachers for the preschoolers' consumption data. Six enumerators were chosen from a team of 10 following the evaluation of their performance in a 5-d training involving theory and field tests. This was done for each round of the survey. Twenty-two class teachers were trained for the exercise in a 3-d workshop at the County Education Offices. The pretests were done with 30 caregivers from Kabondo Kasipul subcounty, an area significantly distant from the study area and equally known for sweetpotato farming. The pretested questionnaires were used to conduct a household-level survey at baseline (in August 2018 before the experiment) and at follow-up (in October–November 2018 after the 30 d of intervention). The surveys collected the households' data on a host of variables including sociodemographics; caregivers' knowledge, attitude, and practices (KAP) regarding OFSP; and institutional factors around food availability and dietary behavior. Data on the caregivers' KAP were elicited using a set of 24 questions in the questionnaire. These were computed to yield a weighted score ranging from 0 to 1, where a higher score implied better KAP.

This study also used an adjusted child's dietary diversity (CDD) measurement tool to limit the food consumption recall period to 24 h instead of 7 d as in the FFQs. This was aimed at addressing the reliability problems in the FFQ by taking care of recall bias and measurement errors due to long recall periods (26). A CDD tool was adjusted from the standard 9 food groups to include 2 extra rows that singled out the “white- and yellow-fleshed sweetpotato” and the “OFSP” foods from the “cereals and other carbohydrates” and the “vitamin A vegetables” food groups, respectively. Thus, the modified tool was labeled a 5-d (school days) dietary diversity register (DDR) (**Supplemental Figure 3**).

The teachers were trained and tested for reliable collection of the preschoolers' food consumption data using the DDR. The teachers sought each preschooler's open recall of what they ate at home over the previous 24 h, on every school day. They recorded the responses on the DDR by checking the boxes on the corresponding food or food group row and within the given date column. Consequently, using the DDR tool, the study captured independent counts of OFSP consumption days among the preschoolers during the intervention period. It captured their 24-h recall consumption data 5 times a week—covering food consumed from Sunday through Thursday—for 4 consecutive weeks in September and October 2018. One of the days (October 10) was a national holiday; thus, the consumption data were collected for 19 d.

The targeted sample was 431 households; however, the baseline survey reached 390 households and the balance was lost to survey declines. Furthermore, a total of 385 complete observations were collected using the DDR and reduced to 373 observations after excluding 12 cases of rampant absenteeism (≥10 d) among the preschoolers. Thus, 17 preschooler-caregiver pairs were treated as experimental dropouts, and there was no significant relation between the study groups and experimental attrition (*P*= 0.308; Fisher's exact tests). Thus, the analysis involved 373 complete observations from the caregivers and the preschoolers.

### Measurement of OFSP consumption days

According to the WHO (27), the frequency of consumption is the number of days of consuming a food or food group over a reference period. The outcome variable, number of OFSP consumption days, was computed as the sum of days when the preschooler consumed an OFSP food as recorded in the DDR. This gave a count variable with values expected to range from 0 to 19.

### Data analysis

The data on the outcome variable, number of OFSP consumption days, were collected for 1 harvesting period in 2018. Thus, they were analyzed as cross-sectional data. The analysis involved 373 observations with covariates drawn from the baseline household-level survey and the outcome variable from the DDR data. First, there was a test for differences in the preschooler, caregiver, and household sociodemographic variables between the control and treatment groups for comparability. Significant mean differences in the number of OFSP consumption days and other variables between the control and each of the treatment arms were also checked using unpaired *t* test and Mann–Whitney *U* test.

The dependent variable is a count variable that measured the number of times OFSP was consumed and hence takes the values 0, 1, 2… *K*. Therefore, count models were considered to regress the number of OFSP consumption days on the intervention variables and other covariates. The standard Poisson regression model (PRM) is the most common model for count data conditions. In this study, we used the zero-inflated Poisson (ZIP) regression technique, which fits better in situations where the dependent variable has zero entries; that is, in the context of our study, where many respondents report that they did not consume OFSP (28, 29).

The ZIP model can be specified as follows:

(1) $$\begin{eqnarray*}
\Pr \left( {\ {Y_i} = {y_i}\ |{x_i}} \right) = \left\{ {\begin{array}{@{}*{2}{l}@{}} {p + \left( {1 - p} \right)\exp \left( { - {\lambda _i}} \right)\ }&{if\ y = 0}\\ {\left( {1 - p} \right)\frac{{{\lambda _i}^{{y_{{i_e}}} - {\lambda _i}}}}{{{y_i}!}}}&{if\ y \textgreater 0} \end{array}} \right.\end{eqnarray*}$$

where ${y_i}$ is the number of OFSP consumption days for a preschooler from household *i*, *x_i_* is a vector of the explanatory variable scores from household *i*, $p$ is the probability of zero OFSP consumption days in the reference period, and $exp( { - \lambda } )$ is the density function of the data-generating process that produces the food frequency score during the intervention period conditioning on the caregiver's decision to prepare the OFSP meals. In the model, $E\ = ( Y )\ = \ \mu \ = ( {1 - p} )\ \lambda $ and $Var\ ( Y ) = \ \mu + \frac{p}{{1 - p}}{\mu ^2}$. The implicit functional form of the ZIP model used to estimate the number of days the preschoolers were fed on OFSP was specified as;

(2) $$\begin{eqnarray*}
&&CONSUMPTIO{N_{ihv}}\ = {\beta _{intervention}}\ INTERVENTIO{N_{ihv}}\nonumber\\
&&\quad +\, {\beta _{child}}CHIL{D_{ihv}} + {\beta _{caregiver}}CAREGIVE{R_{ihvs}}\nonumber\\
&&\quad + \, {\beta _{hh}}H{H_{ihv}} + \ {\beta _{village}}VILLAG{E_{ihv}} + {V_v} + \ {\varepsilon _{ihv}}
\end{eqnarray*}$$

The primary dependent variable (*CONSUMPTION*) is the number of OFSP consumption days for preschooler *i*, in household *h*, that is located in village *v*. In addition to the main focus variables of the nutrition education approaches (*INTERVENTION*), the study controlled for vectors of preschooler- (*CHILD*), caregiver- (*CAREGIVER*), household- (*HH*), and village- (*VILLAGE*) level characteristics. Further, the village cluster fixed effects (*V*) were also controlled for. The symbols *β* and *ε* denote the coefficient estimates and the disturbance term, respectively.

While estimating the ZIP model, potential zero consumption frequencies (zero inflation) were predicted by whether the caregiver cultivated the crop (dummy) and average monthly household expenditure. The model would not converge when more variables were added to predict zero inflation. Also, to overcome a potential estimation problem due to the small sample size—15 clusters and many predictors—the ZIP model estimation space was adjusted by bootstrapping the sample at 1000 replications, as recommended by Long and Freese (28). The study also used a binary logit model to model the likelihood of the preschooler to consume OFSP at a frequency greater than the overall average number of OFSP consumption days in 5 days.

Following the recommendations of Long and Freese (28), the best fitting count model was assessed by comparing the PRM, negative binomial regression model, ZIP, and zero-inflated negative binomial models based on 3 goodness-of-fit statistics: Bayesian information criterion (BIC), Akaike information criterion (AIC), and the likelihood ratio test. The Vuong test was omitted because it is not appropriate for comparing models with zero inflation (30). Overall, the ZIP model had the largest log-likelihood, and smallest AIC and BIC, which implies the best relative goodness-of-fit.

### Ethical review

This study was conducted as per the guidelines laid down in the Declaration of Helsinki. The study was authorized by the Homabay County Early Childhood Development Education Office under reference HBC/EDUC&ICT/PTN/VOL. 1/4/1/8/034 of July 30, 2018. It was also approved by the University of Nairobi's Graduate School under reference A56/89,965/2016. All participating caregivers gave written informed/voluntary consent on behalf of themselves and their preschoolers for their participation in this study.

## Results

### Descriptive statistics

The analysis involved 373 observations from both preschoolers and caregivers, because 17 cases were lost to rampant absenteeism from school among the preschoolers, inter alia. These were distributed as 98, 70, 79, and 126 in the control, PT, IT, and CT arms, respectively. **Table 1** presents the descriptive statistics [mean ± SD and frequency (%)] of the demographics and other socioeconomic characteristics of preschooler-caregiver pairs and their households. It also presents the balancing tests of the covariates (at baseline) in assessing the effectiveness of the randomization in delivering comparable groups. The first column (1) shows statistics for the overall sample. The other columns (2 to 5) present the statistics for the control, PT, IT, and CT groups. The last column (6) presents the *P* values for the test of significant difference in the variables between the 4 study groups. These are the results of 2-factor ANOVA, Kruskal–Wallis, and χ^2^ tests. Results of post hoc multiple-pairwise comparison tests using the Bonferroni correction and the results are presented by the letter superscripts after the values. Same letter superscripts indicate no significant difference in the given variable between the study groups, otherwise significant. At baseline, the groups balanced well in most of the socioeconomic variables except for the caregiver's age, occupation, monthly household expenditures, and CDD. These variables varied differently across the study groups. They were used in the regression models as controls for possible randomization bias.

**TABLE 1:** Description and distribution of variables across the study groups (control, PT, IT, and CT groups)^1^

	1: Total sample	2: Control	3: PT	4: IT	5: CT	6: *P* value^2^
Child and caregiver						
Child's age, y	5.65 ± 1.09	5.78 ± 1.11	5.61 ± 1.08	5.66 ± 1.12	5.56 ± 1.06	0.48
Child's gender (female = 1)	216 (58)	57 (58)	35 (50)	45 (57)	79 (63)	0.39
Caregiver's age, y	35.94 ± 11.51	39.00 ± 12.87^a^	35.09 ± 10.23^ab^	31.96 ± 9.05^b^	36.53 ± 11.78^ac^	0.0008
Caregiver's gender (female = 1)	343 (92)	87 (89)	67 (96)	74 (94)	115 (91)	0.38
Caregiver's education level (post-primary education = 1)	63 (17)	19 (19)	12 (17)	14 (18)	19 (15)	0.86
Caregiver's marital status (married = 1)	310 (83)	79 (81)	58 (83)	69 (87)	105 (83)	0.69
Caregiver's occupation (mainly non-farm = 1)	101 (27)	23 (23)^ab^	14 (20)^a^	16 (20)^a^	47 (37)^b^	0.02
Member of a farmers’ group (yes = 1)	123 (33)	36 (37)	22 (31)	28 (35)	37 (29)	0.57
Caregiver is household head (yes = 1)	153 (41)	45 (46)	25 (36)	31 (39)	53 (42)	0.59
Household (HH)						
HH size (counts)	6.23 ± 2.16	6.40 ± 2.45	6.36 ± 2.09	6.11 ± 1.91	6.10 ± 2.13	0.52
HH has child aged <5 y (yes = 1)	298 (80)	75 (77)	53 (76)	62 (78)	108 (86)	0.24
HH monthly expenditure (KSh × 10^3^)	7.00 ± 7.30	9.37 ± 10.05^a^	6.95 ± 5.90^ab^	5.10 ± 3.45^bc^	6.39 ± 6.81^c^	0.01
Distance to nearest health facility (walking min)	34.33 ± 24.00	38.69 ± 31.73	30.90 ± 15.11	35.63 ± 19.04	32.03 ± 23.51	0.29
Distance to nearest community health volunteer (walking min)	15.06 ± 15.38	16.14 ± 18.64^a^	8.46 ± 7.62^b^	13.96 ± 11.35^a^	18.58 ± 16.85^a^	0.0001
Child dietary diversity score (0–9)	4.32 ± 1.22	4.42 ± 1.29^ab^	4.64 ± 1.05^a^	4.27 ± 1.01^ab^	4.09 ± 1.33^b^	0.02
Child had a diverse diet (yes = 1)	283 (76)	75 (77) ^ab^	63 (90)^a^	61 (77) ^ab^	86 (68)^b^	0.008
Produced OFSP in the first season (yes = 1)	222 (60)	84 (86)^a^	43 (61)^b^	40 (51)^bc^	55 (44)^c^	0.0001
OFSP plot size, m^2^	23.81 ± 45.55	36.44 ± 57.77^a^	26.96 ± 48.22^b^	18.59 ± 41.75^b^	15.51 ± 31.58^b^	0.0001
OFSP consumption knowledge at baseline (0–1)	0.94 ± 0.14	0.94 ± 0.13	0.93 ± 0.16	0.93 ± 0.13	0.94 ± 0.15	0.86
Knowledge of vitamin A at baseline (0–1)	0.65 ± 0.31	0.69 ± 0.29	0.62 ± 0.35	0.66 ± 0.29	0.65 ± 0.31	0.65
Attitude toward OFSP at baseline (0–1)	0.63 ± 0.10	0.62 ± 0.11	0.62 ± 0.11	0.62 ± 0.09	0.64 ± 0.11	0.58
Production and consumption (at endline)						
Grew OFSP to maturity in first season (yes = 1)	209 (56)	80 (82)^a^	40 (57)^b^	38 (48)^b^	50 (40)^b^	0.0001
Preschooler consumed OFSP (yes = 1)	246 (66)	80 (82)^a^	47 (67)^ab^	53 (67)^ab^	67 (53)^a^	0.0002
OFSP consumption frequency (number of days)	1.44 ± 1.51	1.61 ± 1.21^ab^	1.47 ± 1.56^abc^	1.80 ± 1.91^b^	1.06 ± 1.34^c^	0.0009
Consumed at least once per week (1 in 5 d) (yes = 1)	34 (9)	4 (4)^a^	4 (6)^a^	17 (22)^b^	10 (8)^a^	0.0004
Observations	373	98	70	79	126	

1 Data are presented as means ± SDs for continuous variables and *n* (%) for dummy variables. Total sample *n* = 373 for all variables except for OFSP consumption knowledge at baseline (0–1) where *n*  = 369. Control refers to the control group. CT, caregiver treatment group; IT, integrated treatment group; KSh, Kenya shillings; OFSP, orange-fleshed sweetpotato; PT, preschooler treatment group.

2 *P* values show the significance level of mean differences in the variables between the study groups using 2-factor ANOVA and the Kruskal–Wallis test for continuous variables and χ^2^ test for dichotomous variables. Results of post hoc multiple-pairwise comparison, Dunn–Bonferroni tests, are presented by the superscripts. Different superscripts (a, b, c) imply there is a significant difference in the variable between the 2 study groups, otherwise, no significant differences exist.

Source: Survey Data [2018 (this study)].

Overall, the preschoolers were dominantly (58%) girls and averaged 6 y old (range 4–7 y). As expected, a majority of the caregivers were women (92%), with a mean ± SD age of 36 ± 12 y. Also, 4 in 5 households had children aged <5 y. Therefore, the sample suitably captured VAD vulnerable groups (women between 15 and 49 y old and children <5 y old). The average size of the households ranged between 6 and 7, with an average monthly household expenditure of 7000 Kenyan shillings. Only a small minority (17%) of the caregivers had post–primary school education. This reflects the low education standards in rural farming households. Perhaps due to participation in cooking demonstrations and the OFSP vines dissemination activities, the caregivers had good knowledge of OFSP consumption (93.5%), good knowledge of sources of vitamin A and its nutritional benefits (65.2%), and a good attitude toward the food (62.6%). Overall, the baseline consumption knowledge level of the caregivers did not differ across the study groups (Table 1), between the OFSP producers and nonproducers (mean difference = 0.0008; *P* = 0.522) or between households where the preschoolers consumed and did not consume OFSP (mean difference = 0.007; *P* = 0.679). The latter signifies the limited effects of routine OFSP promotion activities, provided during the cultivation period, on consumption of OFSP.

### The relation between OFSP production and consumption trends

**FIGURE 2:**
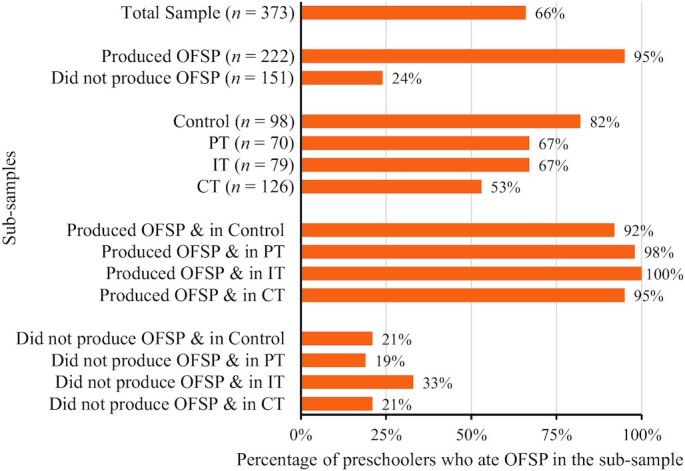
Distribution of consumers of OFSP across the study groups and by OFSP production status. Data are percentages of those who consumed OFSP in the respective subsamples. CT, caregiver treatment group; IT, integrated treatment group; OFSP, orange-fleshed sweetpotato; PT, preschooler treatment group.

A relatively higher proportion of preschoolers consumed OFSP in the control group than the treatment group. The control group also had a significantly higher percentage of those who cultivated the crop than the rest of the groups. This can be linked to their exposure to the free vines dissemination prior to the randomization for the complementary nutrition education interventions of interest. As expected, there was a significant positive relation between the caregivers' production of OFSP and the preschoolers' consumption across all the study groups ( *P* = 0.000; Fisher's exact tests). **Figure 2** provides more insights into the interaction between OFSP cultivation and consumption across the study groups. Overall, 95% (*n* = 209) of households that produced OFSP fed it to their preschoolers. In addition, a significant 24% (*n*   = 164) of households that did not cultivate OFSP also found and fed some to their preschoolers. The latter statistic draws more attention to the food access pattern and what influenced consumption. This could be explained by the traditional culture of sharing sweetpotato root harvests with neighbors and relatives in the rural farming setting of western Kenya. Potentially, these results also point to the power of the intervention approaches in enhancing the consumption of OFSP roots among the social networks of the direct targets. Also note that 21% of the control group consumed OFSP although they did not produce it. Again, this could be due to exposure to the cooking demos implemented prior to the randomization and/or relative proximity to the OFSP producing households who tended to be significantly higher in the control group than the other study groups.

Also, the mean number of OFSP consumption days varied significantly across the study groups (Kruskal–Wallis test; χ^2^ = 17.08; *P* = 0.0007). This was significantly higher in the IT group than in the CT group (*P *= 0.0101), and in the control group than in the CT group (*P* = 0.0004). However, despite all groups recording a mean frequency >1, the IT group treatment was the only approach significantly associated with OFSP consumption for at least once in a 5-d week, on average.

The results further show that although the production of the OFSP was strongly associated with its consumption among preschoolers (Pearson χ^2^ = 198.23; *P* = 0.000), ∼5% of caregivers who produced OFSP did not feed it to their preschoolers. This shows that although production is essential for the availability of OFSP, it does not result in the outright feeding of the preschoolers (Figure 2). These producing but not consuming scenarios were significantly higher in the control and CT groups than in the IT group (Pearson χ^2^ = 4.911; *P* = 0.027 and Pearson χ^2^ = 4.022; *P* = 0.045, respectively).

**FIGURE 3:**
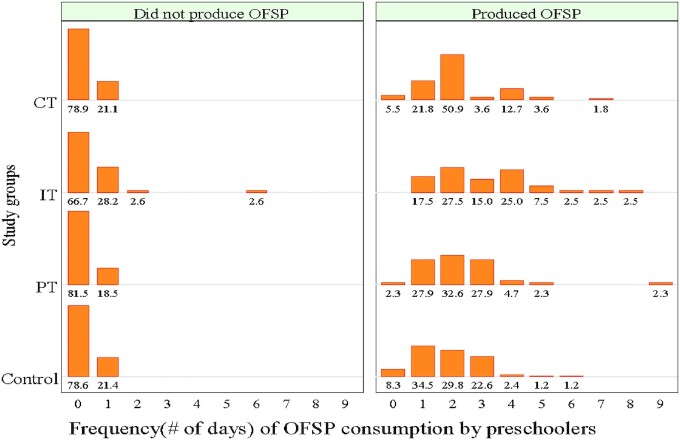
Percentage of preschoolers by OFSP production and number of OFSP consumption days in the study groups. Data are percentages of preschoolers in the respective subsamples (by production and study groups) who consumed OFSP for the different number of days, on the horizontal axis, during the 19 d of food consumption recalls. Did not produce OFSP (*n* = 151); produced OFSP (*n* = 222); control group (*n* = 98). CT, caregiver treatment group (*n* = 126); IT, integrated treatment group (*n*  = 70); OFSP, orange-fleshed sweetpotato; PT, preschooler treatment group (*n* = 73).

**Figure 3** shows the distribution of the preschoolers' consumption frequencies by the study groups and the category of those who produced and those who failed to produce the crop. There were larger proportions of nonconsumers of OFSP in the category of nonproducers, and relatively greater percentages and higher consumption frequencies in the category of producers. It is important to note that the IT group recorded higher percentages in higher consumption frequencies than the other study groups in both categories.

### Effects of nutrition education interventions on OFSP consumption

**Table 2** presents the estimates (coefficients and average marginal effects) of the interventions on consumption, number of OFSP consumption days, and average OFSP weekly consumption days after controlling for child, caregiver, household factors, and village fixed effects. A less parsimonious model is presented in **Supplemental Table 1** for comparison.

**TABLE 2:** The effect of nutrition education interventions on the OFSP consumption and number of consumption days^1^

	1	2	3	4	5	6
	Consume OFSP		Number of OFSP consumption days		Consume more than once per week	
	Binary logit		ZIP		Binary logit	
	Coefficient (95% CI)	dy/dx (95% CI)	Coefficient (95% CI)	dy/dx (95% CI)	Coefficient (95% CI)	dy/dx (95% CI)
Main						
PT	0.55 (−2.47, 3.56)	0.05 (−0.17, 0.27)	0.21 (−0.17, 0.58)	0.27 (−0.27, 0.81)	0.71 (−0.66, 2.09)	0.03 (−0.03, 0.08)
IT	1.23* (−1.94, 4.40)	0.11* (−0.10, 0.33)	0.50** (0.08, 0.91)	0.77** (−0.04, 1.57)	2.66*** (0.79, 4.53)	0.20** (0.01, 0.40)
CT	0.50 (−2.48, 3.49)	0.04 (−0.19, 0.28)	0.16** (−0.06, 0.39)	0.21** (−0.08, 0.50)	1.59** (0.27, 2.92)	0.09** (0.01, 0.16)
Child's age	−0.03 (−0.58, 0.52)	<−0.01 (−0.06, 0.05)	0.01 (−0.08, 0.09)	0.01 (−0.11, 0.13)	0.30* (−0.03, 0.64)	0.02* (−0.00, 0.04)
Caregiver's education	0.38 (−0.41, 1.18)	0.04 (−0.04, 0.12)	0.07 (−0.02, 0.15)	0.10 (−0.03, 0.22)	−0.53 (−1.45, 0.40)	−0.03 (−0.10, 0.03)
Marital status	−0.10 (−1.33, 1.12)	−0.01 (−0.13, 0.11)	0.23* (−0.01, 0.46)	0.33* (−0.03, 0.69)	1.19* (−0.04, 2.42)	0.08* (−0.01, 0.16)
Farmers’ group	0.34 (−0.23, 0.92)	0.03 (−0.03, 0.09)	0.16** (0.01, 0.32)	0.23* (−0.03, 0.49)	0.79 (−0.38, 1.97)	0.05 (−0.02, 0.13)
Distance to nearest CHV (square root)	−0.03 (−0.27, 0.21)	<−0.01 (−0.03, 0.02)	<−0.01 (−0.06, 0.06)	<−0.01 (−0.08, 0.08)	−0.09 (−0.31, 0.13)	−0.01 (−0.02, 0.01)
Produced OFSP	4.41*** (1.48, 7.33)	0.43*** (0.31, 0.56)	1.73*** (1.03, 2.43)	3.05*** (−0.68, 6.78)	3.67*** (2.93, 4.41)	0.24*** (0.17, 0.31)
Monthly expenditure (log)				−0.06* (−0.12, 0.01)		
Constant	−1.65 (−7.42, 4.11)		−4.34*** (−5.53, −3.15)		−9.51*** (−12.36, −6.66)	
Inflate						
Produced OFSP			−24.61*** (−50.95, 1.74)			
Monthly expenditure (log)			2.49 (−20.64, 25.61)			
Constant			−22.23 (−246.12, 201.65)			
χ^2^	66.39***		114.4***		132.4***	
Log-likelihood	−122.9		−464.4		−82.29	
AIC	265.9		954.9		184.6	
BIC	305.1		1005.8		223.8	

^1^The total sample consists of observations from 373 preschoolers who reported to the institutions for ≥11 of the 19 d and gave their food consumption recalls. CIs in parentheses are robust to clustering at village levels. Estimation of the zero-inflated Poisson model was exposed to the variable “present” (the total number of days that the child turned up to the ECD center). All the treatment group variables were compared against the control as base category; levels of significance: *P < 0.10, **P < 0.05, ***P < 0.01. AIC, Akaike information criterion; BIC, Bayesian information criterion; CHV, Community Health Volunteer; CT, caregiver treatment; ECD, Early Childhood Development; IT, integrated treatment; OFSP, orange-fleshed sweetpotato; PT, preschooler treatment. Source: Survey Data [2018 (this study)].

Holding other factors constant, a preschooler whose caregiver is assigned to receive the nutrition education from multiple channels will increase their probability to consume OFSP by 11.5%, and their likelihood to consume OFSP at least once every 5 d by 20.4% relative to a case where no nutrition education is assigned. Again, being in the IT (or CT) groups significantly enhances an individual's OFSP consumption frequency by 76.6% (or 21.2%) compared to a case with no complementary nutrition education at all.

The IT had greater effects on OFSP consumption parameters relative to the CT, whereas PT induced no significant effects. Besides, the IT had stable coefficient and marginal effect estimates between the 2 specifications in Table 2 (and Supplementary Table 1). The negative and positive signs in parameters estimated for zero inflation (OFSP cultivation and the household expenditure) variables, in estimation of the likelihood of nonconsumption of OFSP, are in line with the expectations in previous literature. For instance, sweetpotato consumption is negatively related to household income due to its presumption as a poor man's crop in SSA, and thus labeled as an inferior good (9). However, OFSP consumption was less expected among nonproducers because the households that produced it did not sell it around.

## Discussion

Efforts to promote biofortified crops in rural societies have majored on providing planting materials, training in production and consumption behavior, and nutrition education at production stages. These approaches have been based on the premise of a direct relation between food production, availability, and consumption at the household levels. The findings of this study replicate the conventional wisdom, but with the proviso that not all OFSP-producing households feed it to their preschool children (Figure 2). This challenges the sustainability of the current OFSP promotion approaches; the gap between production and consumption of OFSP can vary due to many factors, including poor postharvest management, seasonality, cost of accessing the roots (31), and time elapsed since initial introduction. For instance, a related study in 3 counties in western Kenya (32) found a similar limitation with cooking demonstrations and field days on the likelihood of the projects’ beneficiaries sharing the vines a year after the projects lapsed.

This study suggests that such gaps could be narrowed by complementary agrinutrition education interventions that target the VAD vulnerable groups through different channels, simultaneously, as demonstrated in this study (Figure 3). The IT approach ensured every household that produced OFSP fed it to the preschoolers. It also influenced the nonproducing households to seek OFSP and feed their children.

The OFSP success story in improving nutrition status is largely grounded on the efficacy trials that have linked its consumption to changes in the vitamin A concentrations in the target's body (33). There is also a growing body of evidence in scaling projects (34) and those that involved multiple types of vitamin A–rich food (35). A critical factor in the efficacy trials is that often they subject the targets to very regular (or daily) consumption of OFSP (7, 36). However, such an intensive consumption behavior does not occur in normal (nonexperimental) household conditions. This emphasizes the need to promote regular consumption of nutritious crops to realize a significant improvement in nutrition outcomes that are commensurate with the results of the efficacy trials. It is also in line with the key recommendation in the Global Nutrition Report (1) emphasizing the need to rethink food systems to ensure that healthy and sustainably produced foods are not only the most accessible and affordable, but are also the desirable choices for all. Previous studies have researched improving the probability of OFSP consumption but produced limited evidence on how to enhance the intensity or frequency of OFSP consumption over time. The findings of a study (15) on the association between knowledge and consumption of OFSP, support the need to provide behavior change communication campaigns. However, it is not guaranteed that any method of behavior change communication would increase consumption.

The results from this study emphasize the role of targeted and integrated nutrition education approaches in improving OFSP consumption behavior. Whereas children will meet their daily dietary requirement for vitamin A on any day that they consume ≥100 g of cooked OFSP roots (6, 7, 11, 37, 38), consumption of the nutritious food on more frequent days is necessary if their nutritional needs are to be met in a regular and stable manner. Therefore, it is imperative to focus on increasing the rate of consumption of OFSP over time to ensure sustainable utilization of the essential micronutrients. From our findings, although the preschooler-oriented nutrition education approach alone induced no significant effects on OFSP consumption, its role in reinforcing the messages delivered through the mobile phones is apparent in the results of the IT approach. Compared with the control, the IT approach significantly increased the consumption of OFSP at all levels. It increased the likelihood of consuming OFSP, the probability of increasing the frequency of its consumption, and the probability of consuming it more than once in 5 d. Therefore, it confirms that a multichannel complementary nutrition education involving preschoolers and caregivers can enhance the likelihood and frequency of OFSP consumption in rural farming households.

Based on our findings, we concluded that there is still a low prevalence of the practice of feeding OFSP to preschool children even in the harvesting periods. Although the production of OFSP assures availability of the nutritious food in the household, complementary nutrition education is needed to encourage consumption of the produce among the preschoolers. Both single- and multichannel nutrition education approaches can be effective in improving the frequency of OFSP consumption. Moreover, phone-mediated text messaging can deliver significant changes in the consumption frequency even when issued as a solitary complementary nutrition education intervention. Contemporary and future agrinutrition education programs should consider both the mobile messaging technology and the preschoolers as effective platforms for promoting regular and sustained consumption of nutritious food crops. Future studies should explore the cost-effectiveness of these single- and multichannel complementary nutrition education interventions involving the ECD platform to solidify the case for their adoption in nutrition programs. The studies could also appraise the interventions to identify factors affecting the variations in their effectiveness and to inform strategic adjustments in agrinutrition programs for sustainable impacts.

A likely limitation of this study is that the preschoolers' consumption data were collected only on school days (5 d/wk) and for 4 wk. There could be considerable variation in household consumption patterns during the missed days. Thus, readers should interpret the results with caution that the OFSP harvesting period might not have been exhaustively covered for some households. Nevertheless, the study employed a very robust implementation approach that involved repeated training sessions for enumerators and class teachers to eliminate chances of response and confirmation biases, and close monitoring of the data collection process to ensure accurate data collection. Further, the innovation of seeking repeated 24-h food consumption recalls from the preschoolers made it possible to collect relatively more accurate and reliable food frequency data, which would not be possible with common tools such as FFQs.

## Supplementary Material

nzab096_Supplemental_File

## Notes

This study was undertaken as part of the Consultative Group on International Agricultural Research Program on Roots, Tubers and Bananas (RTB). Funding for the study was provided by the USAID Feed the Future's Accelerated Value Chain Development (AVCD) project through the International Potato Center (CIP), and the African Economic Research Consortium (AERC) via a research grant offered to SOO, who was a student in the Collaborative Masters in Agricultural and Applied Economics (CMAAE) program. CIP's staff, including JJO and PM, participated in the design and implementation of the study.

Author disclosures: The authors report no conflicts of interest.

Supplemental Figures 1–3 and Supplemental Table 1 are available from the “Supplementary data” link in the online posting of the article and from the same link in the online table of contents at https://academic.oup.com/cdn/.

Present address for PM: HarvestPlus, c/o International Center for Tropical Agriculture (CIAT), International Center for Insect Physiology and Ecology (ICIPE) complex–Kasarani, Nairobi, Kenya.

Abbreviations used: AIC, Akaike information criterion; BIC, Bayesian information criterion; CDD, child's dietary diversity; CIP, International Potato Centre; CT, caregiver treatment; DDR, Dietary Diversity Register; ECD, Early Childhood Development; ECDE, Early Childhood Development and Education; IT, integrated treatment; KAP, knowledge, attitude and practices; OFSP, orange-fleshed sweetpotato; PRM, Poisson regression model; PT, preschooler treatment; SSA, sub-Saharan Africa; VAD, vitamin A deficiency; ZIP, zero-inflated Poisson.
