# Supplementary material for: The Role of Targeted Nutrition Education of Preschoolers and Caregivers on Sustained Consumption of Biofortified Orange-Fleshed Sweetpotato in Kenya
Source: Curr Dev Nutr. 2021 Jul 12;5(8):nzab096. doi: 10.1093/cdn/nzab096 (PMC8357802; doi:10.1093/cdn/nzab096)
Supplement: nzab096_Supplemental_File [file nzab096_supplemental_file.docx]

## Supplemental data

## Supplemental Figure 1: A Sample cover page of OFSP-branded exercise books


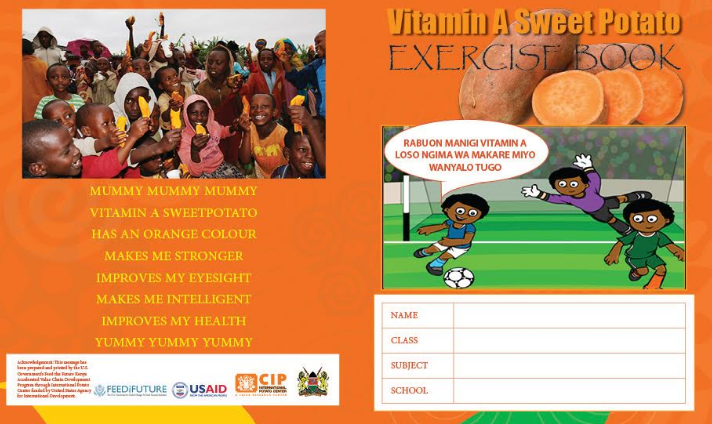


## Supplemental Figure 2: Sample OFSP-branded class posters

| 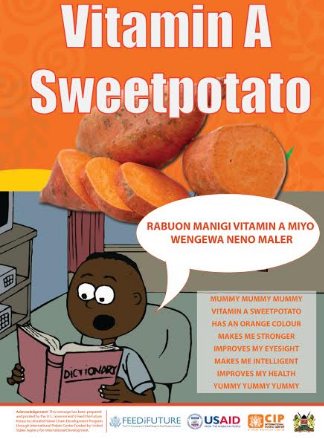 | 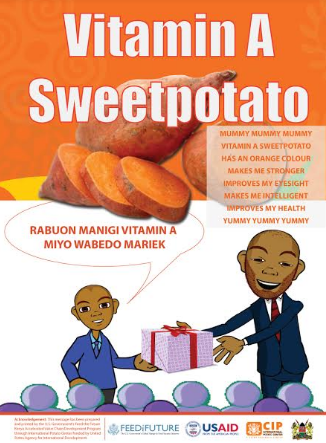 |
| --- | --- |

## Supplemental Figure 3: The adjusted Dietary Diversity Register (DDR)

**
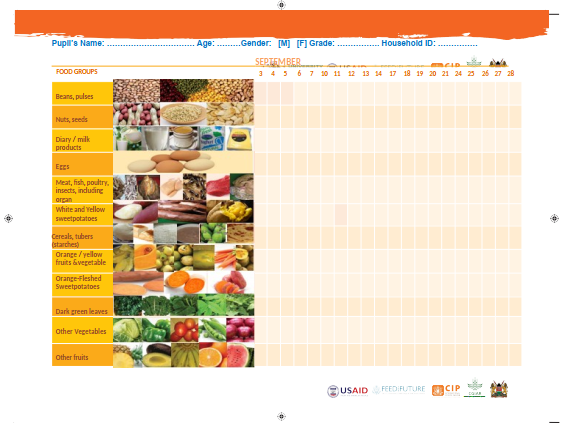
**

**Supplemental Data: Table 1: Estimation of preschoolers’ OFSP consumption data with more covariates**

|  | (1) | (2) | (3) | (4) | (5) | (6) |
| --- | --- | --- | --- | --- | --- | --- |
|  | **Consume** | | **Number of OFSP consumption days** | | **Consume >once per week** | |
|  | **Binary Logit** | | **ZIP** | | **Binary Logit** | |
|  | Coefficient  (95% CI) | dy/dx  (95% CI) | Coefficient  (95% CI) | dy/dx  (95% CI) | Coefficient  (95% CI) | dy/dx  (95% CI) |
| ***Main*** |  |  |  |  |  |  |
| PT | 0.48  (-3.11,4.08) | 0.04  (-0.24,0.32) | 0.20  (-0.18,0.58) | 0.27  (-0.28,0.81) | 0.64  (-1.04,2.31) | 0.02  (-0.04,0.08) |
| IT | 1.06  (-2.66,4.78) | 0.10  (-0.17,0.37) | 0.48***  (0.12,0.85) | 0.74**  (0.04,1.44) | 2.94***  (0.83,5.05) | 0.19**  (0.02,0.36) |
| CT | 0.37  (-3.12,3.86) | 0.03  (-0.26,0.32) | 0.16  (-0.06,0.39) | 0.21  (-0.07,0.48) | 1.92**  (0.07,3.78) | 0.10*  (-0.01,0.20) |
| Child's Age | -0.08  (-0.61,0.46) | -0.01  (-0.06,0.05) | 0.03  (-0.06,0.12) | 0.04  (-0.08,0.17) | 0.41**  (0.03,0.80) | 0.02**  (0.00,0.05) |
| Child's Gender | 0.14  (-0.65,0.93) | 0.01  (-0.07,0.09) | 0.06  (-0.07,0.19) | 0.08  (-0.11,0.28) | -0.65  (-1.46,0.16) | -0.04  (-0.09,0.01) |
| Caregiver's AGE | -0.01  (-0.04,0.03) | <-0.01  (-0.00,0.00) | <0.01  (-0.01,0.01) | <0.01  (-0.01,0.01) | -0.04*  (-0.09,0.01) | <-0.01*  (-0.00,0.00) |
| Caregiver’s Education | 0.44  (-0.64,1.53) | 0.04  (-0.06,0.15) | 0.07  (-0.01,0.15) | 0.10  (-0.03,0.23) | -0.58  (-1.74,0.58) | -0.03  (-0.10,0.03) |
| Caregiver is the HH head | -0.19  (-1.00,0.63) | -0.02  (-0.10,0.06) | 0.02  (-0.15,0.18) | 0.03  (-0.21,0.26) | 0.52  (-0.33,1.36) | 0.03  (-0.02,0.08) |
| Marital status | -0.03  (-1.47,1.41) | <-0.00  (-0.14,0.14) | 0.24  (-0.09,0.58) | 0.35  (-0.15,0.85) | 1.02  (-0.51,2.56) | 0.06  (-0.03,0.15) |
| Farmer group membership | 0.37  (-0.35,1.10) | 0.04  (-0.04,0.11) | 0.17**  (0.02,0.32) | 0.25**  (-0.01,0.50) | 1.09*  (-0.19,2.37) | 0.06  (-0.01,0.14) |
| Knowledge of Vitamin A (Baseline) | 0.17  (-1.39,1.73) | 0.02  (-0.14,0.17) | -0.17  (-0.50,0.15) | -0.25  (-0.74,0.25) | -1.63**  (-2.93,-0.34) | -0.10***  (-0.16,-0.03) |
| Attitude towards OFSP(baseline) | -0.30  (-4.85,4.26) | -0.03  (-0.47,0.41) | 0.24  (-0.43,0.90) | 0.34  (-0.61,1.29) | 0.39  (-4.69,5.48) | 0.02  (-0.27,0.32) |
| HH has under-5-year old | -0.19  (-1.20,0.81) | -0.02  (-0.11,0.08) | 0.15  (-0.05,0.35) | 0.21  (-0.07,0.50) | 0.18  (-1.79,2.15) | 0.01  (-0.10,0.13) |
| Household Size (*square root*) | -0.10  (-1.04,0.84) | -0.01  (-0.10,0.08) | 0.04  (-0.09,0.18) | 0.06  (-0.14,0.26) | 1.35*  (-0.15,2.85) | 0.08*  (-0.00,0.16) |
| Distance to Health Facility (*square root*) | -0.05  (-0.30,0.20) | <-0.01  (-0.03,0.02) | 0.05**  (0.00,0.09) | 0.07*  (-0.00,0.13) | 0.25  (-0.07,0.56) | 0.01  (-0.00,0.03) |
| Distance to Community Health Volunteer(*square root*) | <0.01  (-0.28,0.28) | <0.01  (-0.03,0.03) | -0.01  (-0.06,0.04) | -0.02  (-0.09,0.06) | -0.15  (-0.44,0.14) | -0.01  (-0.03,0.01) |
| Produced OFSP | 4.52***  (1.16,7.89) | 0.44***  (0.29,0.59) | 1.71***  (0.94,2.47) | 3.09  (-1.76,7.94) | 3.87***  (2.83,4.91) | 0.23***  (0.14,0.31) |
| Monthly Expenditure (*log*) | -0.35*  (-0.71,0.01) | -0.03  (-0.07,0.01) | 0.02  (-0.08,0.11) | -0.04  (-0.18,0.11) | 0.12  (-0.61,0.86) | 0.01  (-0.04,0.05) |
| *Constant* | 2.39  (-5.79,10.57) |  | -5.11***  (-6.43,-3.80) |  | -14.06***  (-20.50,-7.62) |  |
| Chi-square (18) | 34.06** |  | 209.7*** |  | 151.8*** |  |
| Log-likelihood | -120.9 |  | -460.5 |  | -72.76 |  |
| AIC | 279.8 |  | 964.9 |  | 183.5 |  |
| BIC | 354.3 |  | 1051.2 |  | 258.0 |  |

*Notes*: Columns (1), (3) and (5) present the coefficient estimates for the respective models (coefficient (95% CI)); columns (2), (4) and (6) present the Average Marginal Effects after estimating the respective models (dy/dx (95% CI)). All model estimates include village fixed effects due to clustering at village levels. The ZIP model estimation was exposed to the variable *present-* (the total number of days that the child turned up to the ECD center/school). The P-values were adjusted using *bootstrap-t* procedure due to few clusters relative to the estimated parameters (15 against 23) ([3](#_ENREF_37)9). All the treatment group variables were compared against the control as base category. The inflating variables are *OFSP Production* and *Monthly expenditure*. PT, Preschooler Treatment; CT, Caregiver Treatment; IT, Integrated Treatment. * p<0.10, ** p<0.05, *** p<0.01. Source: Survey Data (2018)
